# Bioguided Isolation and Structure Identification of Acetylcholinesterase Enzyme Inhibitors from Drynariae Rhizome

**DOI:** 10.1155/2020/2971841

**Published:** 2020-02-23

**Authors:** Ming-Yang Liu, Fan Zeng, Yue Shen, Yu-Ying Wang, Ning Zhang, Fang Geng

**Affiliations:** ^1^Key Laboratory of Photochemistry Biomaterials and Energy Storage Materials of Heilongjiang Province, College of Chemistry & Chemical Engineering, Harbin Normal University, Harbin 150025, China; ^2^College of Jiamusi, Heilongjiang University of Chinese Medicine, Jiamusi, Harbin, Heilongjiang 154007, China; ^3^School of Biomedical Sciences, University of Queensland, St. Lucia, Brisbane 4067, Australia

## Abstract

Drynariae Rhizome, widely distributed in southern China, was clinically used as a traditional treatment for cognitive disfunction, such as Alzheimer's disease (AD). The aim of our work was to evaluate the AChE inhibition activities of extracts of Drynariae Rhizome and pure compounds using a bioguided fractionation procedure. The classical approach for screening potential AChE inhibitors was developed by Ellman. However, the background color of compounds or herb extracts remained uncertain and frequently interfered with the detection of the secondary reaction, thereby easily yielding false positive or false negative results. Here, a high-throughput assay monitoring the transformation of iodized choline from iodized acetylcholine catalyzed by AChE was established based on UPLC-MS/MS. The bioguided fractionation of the extract using this method resulted in the isolation of eight AChE inhibitory flavonoids, including naringenin, eriodictyol, kaempferol, luteolin, astragalin, luteolin-7-O-*β*-D-glucoside, naringin, and neoeriocitrin, with the IC_50_ values of 3.81 ± 0.21 *μ*M, 7.19 ± 0.62 *μ*M, 11.09 ± 1.02 *μ*M, 17.26 ± 0.23 *μ*M, 18.24 ± 2.33 *μ*M, 17.13 ± 1.02 *μ*M, 26.4 ± 1.17 *μ*M, and 22.49 ± 1.25 *μ*M. It is assumed that the identified flavonoids contribute to the AChE inhibition activity of Drynariae Rhizome. These results are in agreement with the traditional uses of Drynariae Rhizome for AD.

## 1. Introduction

According to the “World Alzheimer Report 2018,” Alzheimer's disease (AD) accounts for all types of dementia; nearly, 60–70% of the cases and an estimated 50 million people are suffering with dementia around the world, and this number is forecasted to triple to 152 million by 2025. AD is the most common cause of dementia in senior citizens, which induced a progressive miss of memory and cortical functions which interfere with social and occupational behaviors [1]. Cholinergic neurons play a crucial role in the central and peripheral nervous systems and have been implicated in attention span and cognitive functions [2]. One effective strategy for ameliorating the symptoms of AD is to enhance the acetylcholine (ACh) level *via* inhibiting the acetylcholine esterase enzyme in diseased brain [3]. But the serious side effects caused by licensed drugs used to treat AD have forced researchers to investigate safer acetylcholine esterase enzyme (AChE) inhibitors from natural sources [4, 5]. Numerous plants and their constituents are reported in traditional medicine practices as AChE activity inhibitor to enhance cognitive function and alleviate other symptoms of AD [6–8].

The common method to evaluate AChE activities was developed by Ellman via monitoring the transformation from a substrate acetylcholine (ACh) to the product choline (Ch) catalyzed by AChE for yellow color detection [9]. However, the matrix effect and background color of complex herb extracts is uncertain, often interfering with the detection of the secondary reaction, and easily induces results bias. To improve this fault, the contents of Ch of the reaction system could be determined by a variety of ways, such as thin-layer chromatography [10], fluorimetric assay [11], high-performance liquid chromatography [12], capillary electrophoresis [13], and mass spectrometry [14]. Among these methods, mass spectrometry coupled with ultra-performance liquid chromatography showed advantages in higher sensitivities, high efficiency, better separation, less amount of enzyme, and isotope-label free, which is more suitable for the screening of potential AChE inhibitors from complex systems, such as natural herb extracts [15, 16]. In this study, iodized acetylcholine (ACh-I) and iodized choline iodide (Ch-I) were used as substrate and products instead of nature substances *in vivo*. A triple-quadrupole mass spectrometry (ESI-MS) coupled with an ultra-performance liquid chromatography (UPLC) instrument was involved to detect the activities of potential AChE inhibitors to overcome the complex background of the herb extract.

Drynariae Rhizome, commonly known as Gu-Sui-Bu, is a usual plant widely distributed in southern China [17, 18]. In the past thousands of years, the roots of Drynariae Rhizoma was conventionally regarded as a medicine against osteoporosis and bone resorption, while recently it was increasingly used to treat neurodegeneration diseases, such as AD [19, 20]. In our primary study, the ethanol extract of Drynariae Rhizome showed significant improvement in memory function of aging rats, suggesting that Drynariae Rhizome might contain new AChE inhibitors for AD treatment. In chemical studies, we found that Drynariae Rhizome contains various styles of chemical ingredients, including flavonoids, triterpenes, phenolic acids, and their glycosides, but the biological basis responsible for AChE activity has not been clarified till now [21, 22].

In the present research, the established UPLC-MS/MS method was conducted to isolate the fractionation and purification of the ethanol extracts of Drynariae Rhizome guided by the inhibitory activities of AChE, which resulted in the isolated eight flavonoids as active principles.

## 2. Materials and Methods

### 2.1. Chemicals and Plant Materials

AChE (acetylcholinesterase, obtained from the head of an electric eel, 500 U), iodized acetylcholine (ACh-I, purity > 98.0%), iodized choline (Ch-I, purity > 98.0%), and Tris-HCl buffer (1 mol/L, pH 8.0) were all purchased from Shanghai Source Biotech Co. Ltd. (Shanghai, China). Galantamine hydrobromide (purity > 99.0%) was purchased from Manchester Biotechnology Co. Ltd. (Chengdu, China). UPLC-grade acetonitrile was obtained from Fisher (Fair Lawn, NJ, USA). Water was purified by redistillation and a Milli-Q® ultrapure water system (Millipore, Bedford, MA, USA). Other chemicals and solvents used were of analytical grade.

Drynariae rhizome naturally grown in Jiangxi Province, China, was purchased from Sankeshu Market (DR-20180615, Harbin, China) and authenticated by Professor Ning Zhang, Heilongjiang University of Chinese Medicine.

### 2.2. AChE Inhibitory Activities Assay

AChE inhibition activities of the analytes were determined *in vitro* by monitoring the transformation from a substrate (ACh-I) to the product (Ch-I) catalyzed AChE, and Ch-I was quantified with UPLC-MS/MS to access the AChE activity. The reaction was conducted in a 100 *μ*L mixture, consisting of 50 *μ*L AChE solution (0.5 U/mL), 45 *μ*L ACh-I solution (400 *μ*g/mL), and 5 *μ*L tested sample or blank Tris solution which were all dissolved in Tris buffer (pH 8.0). The mixture was incubated at 37°C in a thermostatic water bath for 25 min and then terminated by 500 *μ*L acetonitrile at 0°C immediately. The terminal solution was centrifuged at 20,000*g* for 15 min, and the supernatant was used for UPLC-MS/MS analysis.

The ACE inhibition was calculated as follows:

(1) $$\begin{array}{c} \text{inhibition }\%=\frac{C_{b}-C_{s}}{C_{b}}*100\%. \end{array}$$

where *C*_*b*_ means the Ch-I concentration incubated without AChE inhibitor and *C*_*s*_ means the Ch-I concentration incubated with inhibitor. The inhibitory activities of the AChE inhibitors were assessed by the IC_50_ values of tested compounds. Galanthamine was selected as a positive control.

### 2.3. UPLC-MS/MS Analysis

Liquid chromatography analysis was accomplished on an ACQUITY UPLC™ system (Waters Corp., Milford, MA, USA). The chromatographic separation was carried out using an ACQUITY UPLCTM BEH C18 column (2.1 × 50 mm, 1.7 *μ*m) at 30°C. The isocratic elution mobile phase consisted of 0.5% acetic acid (A) and acetonitrile (B) (40 : 60) to guarantee the peak shape of Ch-I.

A Waters® Micromass® Quattro Premier™ XE triple-quadrupole tandem mass spectrometer equipped with an electrospray ionization interface (TQ-ESI) was used for mass spectrometric detection. The positive ESI source operation optimal parameters were capillary voltage 3.0 kV, extractor voltage 3.00 V, skimmer 25.0 V, fragmentor 35 V, octopole Rf peak 3000 v, source temperature 120°C, and desolvation temperature 300°C. Nitrogen was used as the desolvation gas and cone gas at a flow of 600 L/h and 50 L/h, respectively. Argon was used as the collision gas at a pressure of approximately 2.61 × 10^−3^ mbar. A multiple reaction monitoring (MRM) mode was used to quantify Ch-I, and the fragments of 104.34 Th and 58.53 Th were selected as the parent ion and daughter ion to obtain accurate quantitation in the mixture containing herb extract. The MS scan rate spectrum was 5000 amu/s, dwell time 5 ms, and *m/z* range 0–300 Th. All data were acquired and analyzed by Mass Lynx™ NT 4.1 software with the Quan Lynx™ program (Waters, Milford, MA, USA).

### 2.4. Method Validation

The linear relationship of the assay was studied by nine different concentrations of Ch-I in Tris-HCl buffer solution at the range of 2 ng/mL–20 *μ*g/mL. The limit of detection (LOD) and the limit of quantification (LOQ) were the concentration giving a signal-to-noise (S/N) ratio of at least 3-fold and 10-fold, respectively. The intraday and interday precisions were investigated by determining quality control samples at three different concentrations (*n* = 6) in three consecutive days. The repeatability was evaluated with the Ch-I concentration in nine incubated systems repeatedly. Stability studies were also investigated at three levels of the QC samples previously described (*n* = 6), which were stored for 24 h at ambient temperature (25 ± 2°C) and three days at 4°C. The precision, repeatability, and stability were expressed with relative standard deviation (RSD). The accuracy of the assay was expressed by the recovery of the quality control samples at three concentration levels. The recovery of analytes at three levels (0.02 *μ*g/mL, 0.1 *μ*g/mL, 1 *μ*g/mL, *n* = 6) was estimated by spiking high, medium, and low concentrations of Ch-I into the reaction solution without AChE. The matrix effect was determined by comparing the MRM peak responses of the extract of Drynariae rhizome (*n* = 6) in enzymes (pretreatment with acetonitrile) devitalized to those of the same analyte presented in the initial mobile phase (*n* = 6).

### 2.5. Bioguided Extraction and Isolation Procedure

**Figure 1:**
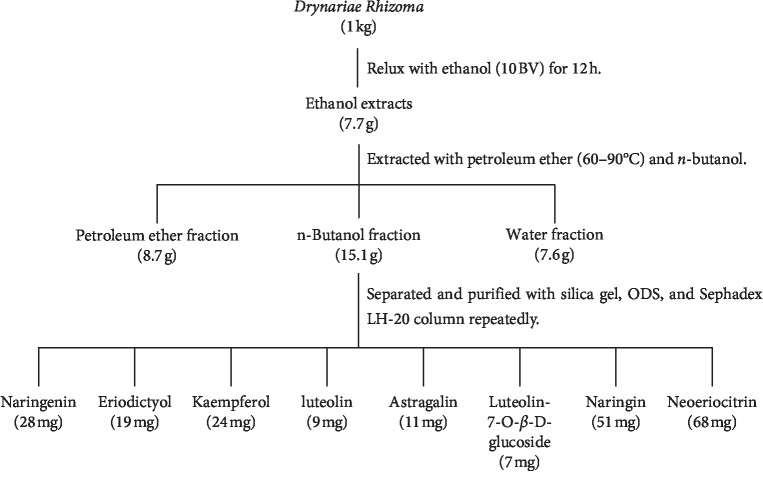
Isolation schedule of Drynariae Rhizoma fractions and ingredients guided by active AChE inhibitory activities.

To acquire the potential inhibitory components from the roots of Drynariae Rhizome, the fractionation procedure was carried out as shown in Figure 1. The powdered air-dried (1 kg) Drynariae Rhizome was extracted with 80% ethanol under reflux for three times (8 BV, 2 hours each time). After filtration, the 80% ethanol solvent was evaporated in vacuum, and the aqueous residue was partitioned successively with petroleum ether (60–90°C) and *n*-butanol. The 80% ethanol extract of Drynariae Rhizome, petroleum ether fraction (8.7 g), *n*-butanol fraction (15.1 g), and water fraction (7.6 g) were tested for AChE inhibition activity by monitoring the conversion of substrate ACh-I to the product Ch-I using this UPLC-MS/MS analysis. The *n*-butanol fraction showed stronger activity with an IC_50_ value of 5.62 mg/mL, compared to the petroleum ether and water fractions with the IC_50_ value of 35.48 mg/mL and 36.31 mg/mL. Therefore, the *n*-butanol fraction was further separated with silica gel, ODS, and Sephadex LH-20 gel repeatedly, from which eight individual flavonoids were obtained and identified finally.

## 3. Results and Discussion

### 3.1. Optimization of AChE Reaction Procedure

**Figure 2:**
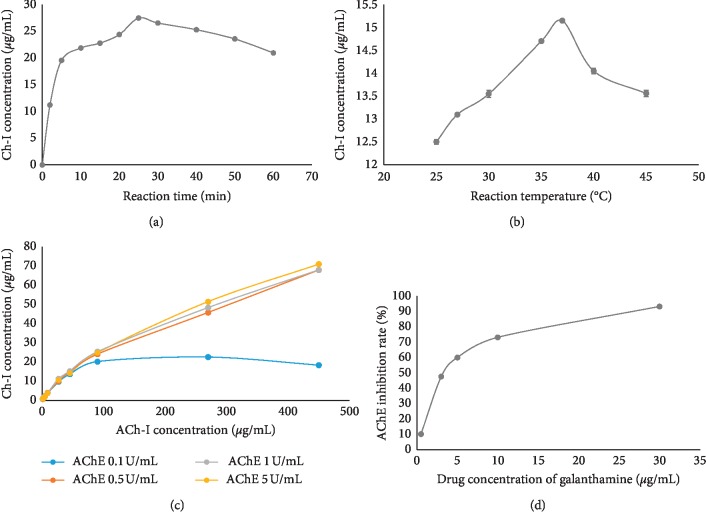
Optimum reaction parameters of AChE-catalyzed reaction. (a) Time course of production of Ch-I converted from ACh-I. (b) Effect of reaction temperature on product of Ch-I. (c) Optimum concentration of enzymes and substrate. (d) AChE inhibition rate of galanthamine.

**Figure 3:**
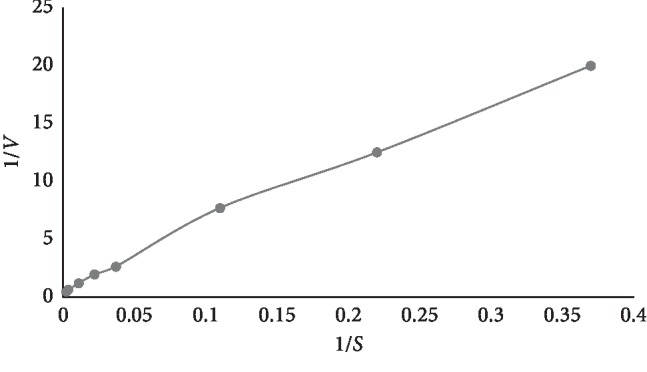
Double reciprocal mapping for the Km value of acetylcholinesterase.

The reaction procedure was conducted with reference to Liu's report [14]; moreover, the enzyme constants, steady-state kinetics, reaction temperature, reaction time, substrate concentration, and AChE concentration were investigated and optimized to guarantee the utilization. As shown in Figure 2, the optimal reaction time and temperature were 25 min and 37°C for the enzyme activity maximization. The concentration of AChE in Liu's report [14] was 3.470 U/mL. But we compared the AChE of different concentrations at 0.5 U/mL, 1 U/mL, and 5 U/mL, resulting in similar catalysis. Thus, 0.5 U/mL was selected as the optimal concentration of AChE. Acetonitrile at 0°C demonstrated better repeatability than methanol and 1M HCL in terminating enzyme reaction. At the given concentration of AChE in reaction solution, the starting concentration of ACh-I was investigated, and 400 *μ*g/mL was selected, which matched the Km value (Figure 3) [23]. The positive control galantamine was used to evaluate the AChE inhibitory activity assay with the IC_50_ value of 1.26 ± 0.15 *μ*M [24].

### 3.2. UPLC-MS/MS Conditions and Method Validation

**Figure 4:**
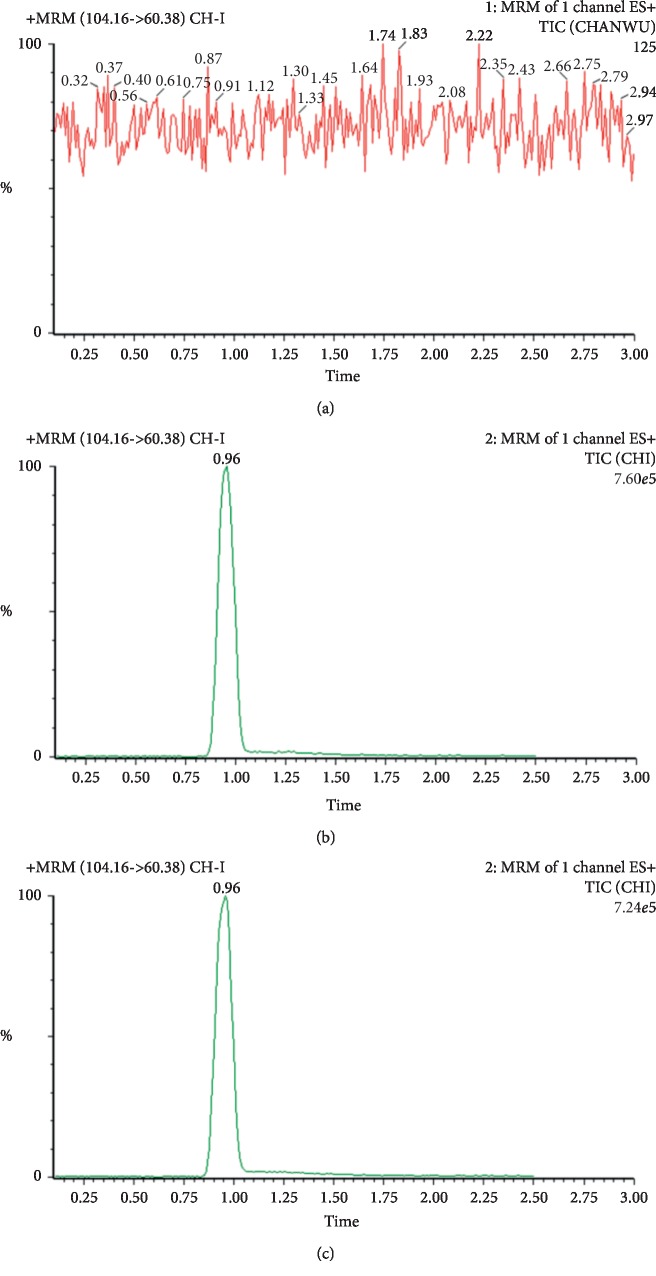
AChE activity determination by UPLC-MS/MS. Representative MRM chromatograms of (a) blank mixture incubated without the substrate, (b) a spike of Ch-I in a tris-buffer, and (c) Ch-I in the sample after the enzymatic reaction.

The MRM mode quantitation method was involved to evaluate the linearity, precision, stability, and accuracy (Figure 4). The calibration showed a linear behavior over a range of 2 ng/mL–20 *μ*g/mL (*r*^2^ = 0.9991). The LOD (S/N = 3) of Ch-I was 8 pg, and the LOQ (S/N = 10) was 17 pg. The precision of the method was assessed at three different concentration levels by calculating the intraday and interday variations of six replicates. All of the precision RSD% of Ch-I was in the range of 1.04%–3.99% (*n* = 6). Ch-I in the reacted mixture was stable after being placed at room temperature (25°C) for 24 h and kept in a refrigerator (4°C) for three consecutive days. The repeatability RSD% of the AChE reaction mixture was 3.57% (*n* = 6). The average accuracies were 97.25, 99.48, and 101%, with the RSD% of 3.14, 2.05, and 3.29% for the high, medium, and low concentrations, respectively. The matrix effects ranged from 89.00 to 109.03%.

### 3.3. Comparison of UPLC-MS/MS Method and Spectrometry Method

A comparative study between this UPLC-MS/MS method and the classic spectrometry method was performed. We analyzed the inhibitory activity of galantamine and extract of Drynariae Rhizome via this novel method and modified Ellman's method [23]. We found that both assays displayed identically dose-dependent effects of the two inhibitors on AChE activity. IC_50_ values of galantamine and extract of Drynariae Rhizome from UPLC-MS/MS assay were 1.26 ± 0.15 *μ*M and 8.21 ± 1.36 *μ*g/mL, which were lower than 1.85 ± 0.45 *μ*M and 24.31 ± 6.90 *μ*g/mL from Ellman's assay. It is noticeable that the UPLC-MS/MS assay directly detects product Ch-I in the MRM mode, whereas the Ellman assay indirectly measures the product through employing a secondary reaction. The background color of the herb extract yielded to a significant positive result, indicating that our UPLC-MS/MS method showed more accuracy and reliability in AChE activity analysis.

### 3.4. AChE Inhibitory Activities of Drynariae Rhizome Extracts and Bioguided Isolation

In order to isolate the compounds responsible for AChE inhibitory, a bioguided fractionation strategy was performed throughout the procedure (Figure 2). The AChE inhibitory activities (IC_50_ values) of petroleum ether, *n*-butanol, and water fractions were summarized in Table 1. Among the three fractions, *n*-butanol fraction showed the most potent AChE inhibitory (5.62 ± 0.23 *μ*g/mL). *n*-butanol fraction was further separated by silica gel, ODS, and Sephadex LH-20 gel, repeatedly, resulting in the obtainment of eight pure compounds. All of the eight compounds were all obtained as yellow amorphous powder and verified to be flavonoids.

**Figure 5:**
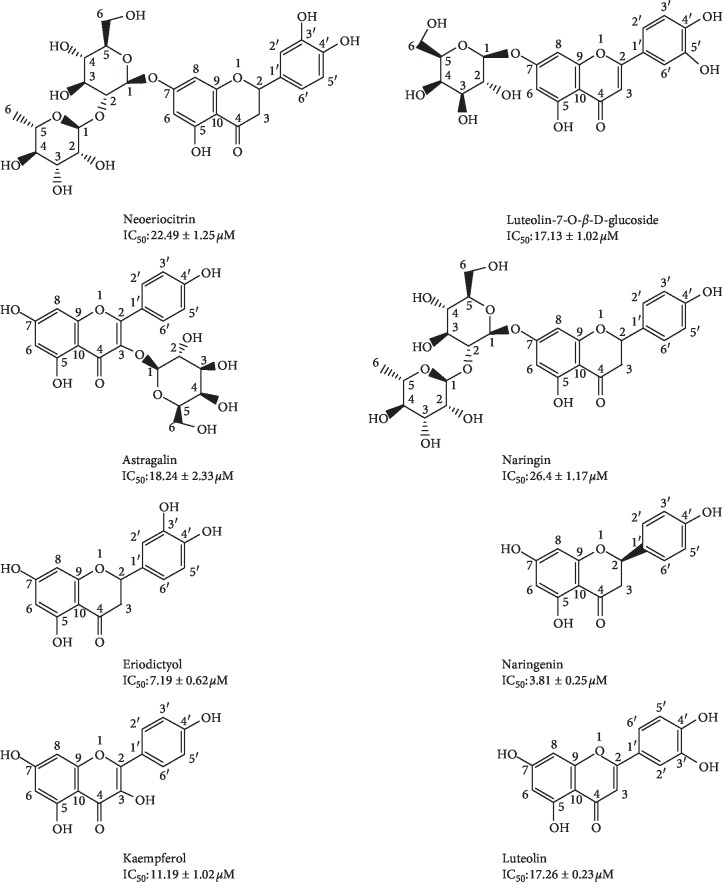
Chemical structures of the eight flavonoids.

Compared with the reported data [25–28], the eight compounds were flavonoids and identified as naringenin (28 mg), eriodictyol (19 mg), kaempferol (24 mg), luteolin (9 mg), astragalin (11 mg), luteolin-7-O-*β*-D-glucoside (7 mg), naringin (51 mg), and neoeriocitrin (68 mg) by UV, IR, MS, ^1^H-NMR, ^13^C-NMR, and 2D-NMR. The ^13^C-NMR data of these compounds are given in Table 2. The chemical structures are given in Figure 5.

### 3.5. AChE Inhibitory Activity of the Eight Flavonoids

The AChE inhibitory activities of the eight flavonoids, including naringenin, eriodictyol, kaempferol, luteolin, astragalin, luteolin-7-O-*β*-D-glucoside, naringin, and neoeriocitrin, were evaluated *in vitro* and summarized in Table 3 and Figure 5. As all the eight flavonoids own the resembling frame structure of flavonoids, a class of yellow pigments derived from flavone (2-phenylchromone) as mother nucleus showed similar AChE inhibitory activity measured by IC_50_ values. Naringenin and eriodictyol are flavanone aglycones, showing the highest AChE inhibitory activities with the IC_50_ values of 3.81 ± 0.21 *μ*M and 7.19 ± 0.62 *μ*M. As flavonoid aglycones, the IC_50_ values of kaempferol and luteolin were measured as 11.09 ± 1.02 *μ*M and 17.26 ± 0.23 *μ*M. We found that the four flavonoid glycosides, astragalin, luteolin-7-O-*β*-D-giucoside, naringin, and neoeriocitrin showed lower AChE inhibitory activities than flavonoid aglycones, differing in the number of glycosides. Astragalin and luteolin-7-O-*β*-D-giucoside are flavonoid monoglycosides with the IC_50_ values of 18.24 ± 2.33 *μ*M and 17.13 ± 1.02 *μ*M, while naringin and neoeriocitrin are diglycosides with the higher IC_50_ values of 26.4 ± 1.17 *μ*M and 22.49 ± 1.25 *μ*M.

## 4. Conclusion

A UPLC-MS/MS method for the determination of the inhibitory activity of AChE inhibitors was developed using ACh-I as the substrate. This high-throughput screening assay for potential AChE inhibitors from natural medicinal plants is more efficient, more sensitive, and lower cost compared with the conventional methods. Eight AChE inhibitors were obtained from Drynariae Rhizoma guided by the AChE inhibition, concluding that the eight flavonoids could be the lead compounds for suppressing the inactivation of acetylcholine and ameliorating symptoms induced by neurodegeneration in AD patients.

## Figures and Tables

**Table 1:** Inhibitory activity (IC_50_) of the fractions investigated against AChE. (*n* = 6).

Fractions	IC_50_ (*μ*g/mL ± SD)
Ethanol extract	8.21 ± 1.36
n-Butanol fraction	5.62 ± 0.23
Water fraction	36.31 ± 3.68
Petroleum ether fraction	35.48 ± 9.93

**Table 2:** ^13^C-NMR data (500 MHz, CD_3_OD) of the eight flavonoids (ppm).

Position	Naringenin	Eriodictyol	Kaempferol	Luteolin	Astragalin	Luteolin-7-O-*β*-D-glucoside	Naringin	Neoeriocitrin
1								
2	79.1	78.·	146.4	164.3	156.8	164.5	80.5	79.3
3	42.6	41.9	135.7	102.9	133.2	103.0	44.0	42.9
4	196.3	196.0	175.9	181.7	177.2	181.8	198.3	197.2
5	164.3	163.4	160.7	157.6	161.2	161.1	165.5	163.6
6	95.8	94.9	98.2	99.2	99.6	99.5	96.5	96.4
7	166.4	166.6	163.7	164.5	165.6	162.9	166.4	165.2
8	95.1	95.7	93.5	94.0	93.9	94.7	95.3	95.4
9	163.5	162.4	156.0	161.5	156.1	156.9	164.4	163.2
10	102.3	101.8	103.1	103.7	103.5	105.3	104.7	103.5
1′	129.9	129.4	121.7	121.5	120.1	120.9	130.6	130.1
2′	128.1	114.3	129.5	113.9	130.1	113.3	128.9	113.4
3′	115.0	145.7	115.1	146.3	115.2	145.9	116.1	145.1
4′	157.6	145.0	159.2	149.9	160.4	150.5	158.9	145.6
5′	115.3	115.3	115.1	116.1	115.8	115.9	116.1	114.8
6′	128.1	117.9	129.6	119.2	130.3	119.2	128.9	118.0
1″	—	—	—	—	—	99.9	—	—
2″	—	—	—	—	—	73.1	—	—
3″	—	—	—	—	—	76.4	—	—
4″	—	—	—	—	—	70.8	—	—
5″	—	—	—	—	—	82.0	—	—
6″	—	—	—	—	—	60.6	—	—
Glc-1	—	—	—	—	101.1	—	102.4	101.2
Glc-2	—	—	—	—	74.3	—	79.0	77.8
Glc-3	—	—	—	—	77.5	—	78.8	77.6
Glc-4	—	—	—	—	69.9	—	71.0	70.8
Glc-5	—	—	—	—	76.5	—	77.9	76.7
Glc-6	—	—	—	—	60.9	—	62.0	60.9
Rha-1	—	—	—	—	—	—	99.1	98.0
Rha-2	—	—	—	—	—	—	72.0	70.8
Rha-3	—	—	—	—	—	—	72.0	69.8
Rha-4	—	—	—	—	—	—	73.7	72.5
Rha-5	—	—	—	—	—	—	69.8	68.6
Rha-6	—	—	—	—	—	—	18.0	16.9

**Table 3:** Inhibitory activity (IC_50_) of the tested compounds investigated against AChE (*n* = 6).

Compounds	IC_50_ (*μ*M ± SD)
Galantamine	1.26 ± 0.15
Naringenin	3.81 ± 0.21
Eriodictyol	7.19 ± 0.62
Kaempferol	11.09 ± 1.02
Luteolin	17.26 ± 0.23
Astragalin	18.24 ± 2.33
Luteolin-7-O-*β*-D-glucoside	17.13 ± 1.02
Naringin	26.4 ± 1.17
Neoeriocitrin	22.49 ± 1.25

## Data Availability

The data used to support the findings of this study are available from the corresponding author upon request.
